# The impact of heart rate on echocardiographic measures of left ventricular function: novel insights facilitated by deep learning

**DOI:** 10.1093/ehjimp/qyaf163

**Published:** 2025-12-24

**Authors:** Ada Woelfert, Ole Christian Mjølstad, Ane Cecilie Dale, Øyvind Salvesen, Lasse Lovstakken, Håvard Dalen, Andreas Østvik, Bjørnar Grenne

**Affiliations:** Department of Circulation and Medical Imaging, Norwegian University of Science and Technology, Box 8905, Trondheim 7491, Norway; Department of Circulation and Medical Imaging, Norwegian University of Science and Technology, Box 8905, Trondheim 7491, Norway; Clinic of Cardiology, St. Olavs University Hospital, Trondheim, Norway; Department of Circulation and Medical Imaging, Norwegian University of Science and Technology, Box 8905, Trondheim 7491, Norway; Clinic of Cardiology, St. Olavs University Hospital, Trondheim, Norway; Department of Public Health and Nursing, Norwegian University of Science and Technology, Trondheim, Norway; Department of Circulation and Medical Imaging, Norwegian University of Science and Technology, Box 8905, Trondheim 7491, Norway; Department of Circulation and Medical Imaging, Norwegian University of Science and Technology, Box 8905, Trondheim 7491, Norway; Clinic of Cardiology, St. Olavs University Hospital, Trondheim, Norway; Levanger Hospital, Nord-Trøndelag Hospital Trust, Levanger, Norway; Department of Circulation and Medical Imaging, Norwegian University of Science and Technology, Box 8905, Trondheim 7491, Norway; Clinic of Cardiology, St. Olavs University Hospital, Trondheim, Norway; Medical Technology, Health Research, SINTEF Digital, Trondheim, Norway; Department of Circulation and Medical Imaging, Norwegian University of Science and Technology, Box 8905, Trondheim 7491, Norway; Clinic of Cardiology, St. Olavs University Hospital, Trondheim, Norway

**Keywords:** echocardiography, deep learning, heart rate, atrial pacing, global longitudinal strain, ejection fraction

## Abstract

**Background:**

Echocardiographic measurements of the left ventricle (LV) are fundamental in diagnosing and monitoring cardiac disease. Still, current understanding of how heart rate influences these measurements is incomplete. We aimed to explore the relationship between heart rate and LV global longitudinal strain (GLS), ejection fraction (LVEF), end-diastolic (LVEDV), and end-systolic volumes (LVESV), using atrial pacing and a transparent multi-step deep learning (DL)-based method for fully automated measurements.

**Methods and results:**

Fifty participants with permanent pacemakers were enrolled. Heart rate was increased by atrial pacing in increments of 10 beats/min, from 50 to 140 beats/min, with echocardiographic 10-beat cine-loops recorded at each step. A DL-based method was utilized to measure GLS, LVEF, LVEDV, and LVESV at all levels.

A total of 10 161 heart cycles were analysed, with 97% feasibility. As heart rate increased, all LV measures displayed significant and near-linear reductions. From 60 to 140 beats/min, GLS decreased by 32% (95% CI: 19–44%), LVEF by 33% (95% CI: 19–47%), LVEDV by 31% (95% CI: 19–43%), and LVESV by 10% (95% CI: −5% to 24%). Processing time per cardiac cycle was 1.3 (0.4) s, corresponding to 3.7 h for the entire dataset.

**Conclusion:**

Heart rate significantly influences echocardiographic measures of LV function and volume, emphasizing the necessity of incorporating heart rate into clinical interpretation and reporting of echocardiographic measurements. This study further demonstrates the potential of DL to advance cardiovascular research by enabling rapid, accurate, and reproducible analyses, previously unachievable due to the inherent constraints of manual measurements.

## Central Illustration

**Central Illustration:**
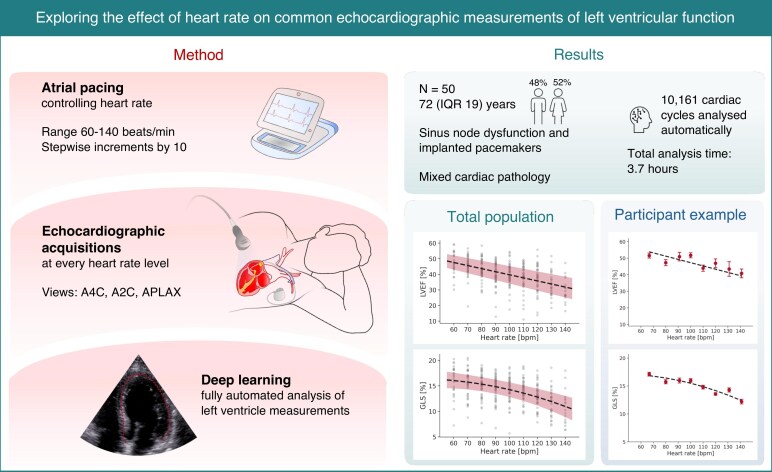
This study aimed to explore the effect of heart rate on common echocardiographic measurements of left ventricular function using atrial pacing and a deep learning-based method. Deep learning enabled fully automated beat-to-beat echocardiographic analysis of 10 161 cardiac cycles. Findings reveal significant heart rate–dependent changes in key LV measures, emphasizing the need to account for HR in clinical echocardiography. A4C, apical four-chamber; A2C, apical two-chamber; APLAX, apical long axis; IQR, interquartile range; LVEF, left ventricular ejection fraction; bpm, beats per minute; GLS, global longitudinal strain.

## Introduction

Echocardiography is a cornerstone in the evaluation of left ventricular (LV) function,^1^ yet challenges arise when interpretating common echocardiographic variables in patients with varying heart rates. The relationship between heart rate and measures of cardiac function has intrigued clinicians and researchers for decades,^2–4^ and understanding this interplay is crucial for accurately assessing cardiac function across a wide range of heart rates. Normal values have been established for variables such as LV global longitudinal strain (GLS), LV ejection fraction (LVEF), and LV volumes, but still, these values predominantly derive from patients within a limited heart rate range.^5,6^ Although those reference ranges are shown to be associated with heart rate,^6^ and recommendation papers advocate reporting heart rhythm during echocardiography,^7^ the influence of heart rate on these key echocardiographic measurements remains inadequately explored.

Despite its scientific relevance, methodological obstacles have precluded comprehensive analyses of the relationship between heart rate and LV function parameters. Alterations in LV measurements induced by changes in heart rate could be subtle and potentially masked by random beat-to-beat variability. Averaging measurements across multiple cardiac cycles offers a methodological solution but has been unachievable due to the time-consuming demands of manual or semi-automatic measurements. Recent advances in echocardiography based on deep learning (DL) now allow for accurate and fully automated measurements of GLS, LVEF, and LV volumes, with improved reproducibility compared to existing semi-automatic methods.^8,9^ Utilizing this capacity to process extensive datasets with tremendous speed and without human input, DL-based measurements averaged across multiple cardiac cycles can reduce random variability, offering research opportunities previously deemed unattainable.

The aim of this study was to explore the relationship between heart rate and echocardiographic measurements of GLS, LVEF, and LV volumes, using DL-based fully automated measurements over multiple cardiac cycles in participants with permanent pacemakers and controllable heart rate by atrial pacing.

## Methods

### Study design and population

We prospectively enrolled 50 participants with permanently implanted programmable pacemakers with right atrial leads, all scheduled for routine outpatient pacemaker follow-up. All participants had isolated sinus node dysfunction with presumed sustained atrio-ventricular (AV) conduction. Inclusion criteria were age ≥18 years and the ability to provide informed consent. Exclusion criteria were any AV or bundle branch block at baseline, ongoing arrhythmia, and decompensated heart failure. Baseline characteristics, including age, height, and body mass index, other cardiac diseases or risk factors, and the use of anti-arrhythmic drugs, were registered based on information directly from the study participants and from the electronic medical records.

After routine interrogation of the pacemaker, a comprehensive echocardiographic examination was performed at baseline heart rate. Echocardiograms at baseline were acquired without pacing if sinus base rate was ≥50 beats/min, otherwise atrial pacing rate was set to 50 or 60 beats/min, depending on the participants individual pacemaker settings. Baseline registrations were followed by a cardiac pacing protocol including stepwise increments in heart rate, with corresponding echocardiographic acquisitions at each step (*Central Illustration*). No participants were excluded based on image quality.

### Cardiac pacing protocol

Cardiac pacing was done in AAI mode, implicating only atrial pacing without any ventricular backup. The target heart rate range was 50–140 beats/min. In participants with sinus rhythm above the pacing threshold, the baseline was determined by the participant’s spontaneous heart rate; otherwise the baseline was determined by the pacemaker setup. The heart rate was thereafter increased by atrial pacing in stepwise increments of 10 beats/min/level, beginning at a whole 10. Echocardiographic acquisitions were obtained immediately after stabilization of the heart rate at each level, before proceeding to the next pacing rate. If participants developed more than first-degree AV block, frequent atrial or ventricular ectopic activity, or experienced uncomfortable palpitations or other significant side effects, the protocol was terminated. Otherwise, the protocol was continued until a heart rate of 140 beats/min was achieved.

### Echocardiographic acquisitions

Echocardiographic images at baseline were obtained as recommended by the European Association of Cardiovascular Imaging (EACVI) and the American Society of Echocardiography (ASE).^10^ Images were acquired with the patient in the left lateral decubital position, using a Vivid E95 scanner (GE Healthcare, Horten, Norway) equipped with a 4Vc-D phased-array transducer. All examinations were performed by experienced cardiologists (BG or HD, both EACVI certified in transthoracic echocardiography, >10 000 acquisitions and measurements) at the EACVI-accredited echocardiographic laboratory at Clinic of Cardiology, St. Olavs University Hospital, Trondheim, Norway. Great care was taken to ensure standardized images.

The echocardiographic protocol involved acquisitions of multi-beat cine-loops at each pacing level, each consisting of 10 consecutive cardiac cycles for each of the three standard apical views: apical four-chamber (A4C), apical two-chamber (A2C), and apical long-axis (APLAX). Recordings were acquired during breath-hold or quiet breathing. The echocardiograms were digitally stored in Raw DICOM (Digital Imaging and Communications in Medicine) file format.

### Manual echocardiographic measurements

The baseline echocardiograms were manually analysed by an experienced cardiologist (BG), using EchoPAC SWO version 206 (GE Healthcare). Measurements were performed as recommended by the EACVI and ASE,^10^ and included GLS by the Automated Function Imaging method, LVEF, LV end-diastolic volume (LVEDV), and LV end-systolic volume (LVESV) by the Simpson’s biplane method, mitral *E*/*A* ratio, *E*/*e*′ ratio, tricuspid annular plane systolic excursion, and lateral tricuspid annular systolic velocity. For the manual measurements, the most representative cycle with best image quality for each relevant view was selected. Furthermore, image quality was scored on a segmental basis at baseline for the three standard apical views, using an 18-segment LV model. Segments were scored as missing if <50% of segmental myocardium was visualized. Overall image quality was considered as good if no segments were missing, intermediate if 1 segment was missing, and poor if ≥2 segments were missing.

### DL-based fully automated echocardiographic measurements

A fully automated multi-step DL-based method was used to measure GLS, LVEF, LVEDV, LVESV, and mitral annular plane systolic excursion (MAPSE) for all cardiac cycles at all heart rate levels, requiring no manual operator input. The DL method has been developed by our group, and technical details have been published previously.^8,11–14^ The method is composed of four artificial neural networks: (i) view classification; (ii) event timing; (iii) image segmentation; and (iv) motion estimation. The view classification network automatically identifies and categorizes the A4C, A2C, and APLAX views, while the event timing network identifies end-diastolic (ED) and end-systolic (ES) frames directly from the B-mode images. Subsequently, the segmentation network classifies the pixels in the cardiac images into four classes: LV myocardium, left atrium, lumen, and background/other. This segmentation enables delineation of the LV myocardial border and the placement of tracing points along the myocardium, in addition to the mitral annulus points. Finally, the motion estimation network is used to propagate the positions of tracing points frame-by-frame throughout the cardiac cycle to calculate GLS, LVEDV and LVESV, and MAPSE. GLS was calculated as the Lagrangian peak strain and presented as an absolute value. LVEDV and LVESV were calculated using the Simpson’s biplane method, and further used to estimate LVEF and cardiac output (CO). MAPSE was calculated as the distance between the mitral annulus points in ED and ES, and the final value was obtained by averaging across all available views. As a comprehensive validation of the automatic MAPSE measurement is currently ongoing, MAPSE is presented here as a secondary result.

The DL method analysed all 10 cardiac cycles within each cine-loop, and per-view average measurements were calculated. These per-view means were then averaged across all relevant echocardiographic views for each specific measurement within each participant. Thus, GLS at each heart rate level was averaged across the 10-cycle per-view means for the A4C, A2C, and APLAX views. Correspondingly, LVEDV, LVESV, LVEF, and CO were calculated based on the 10-cycle means of the A4C and A2C views.

### Quality control and feasibility assessment

The multi-step DL method allows a high level of transparency, allowing implementation of a quality assurance process. Continuous plots were constructed for GLS, LVEDV, and LVESV for each individual study participant. Outliers were controlled manually by reviewing the corresponding multi-beat cine-loops. Measurements were excluded if calculations were based on misclassified echocardiographic views. Feasibility was later assessed for the DL-based method as the proportion of cardiac cycles with accepted measurements.

### Statistical analysis

Echocardiographic data within each heart rate level were inspected visually with QQ-plots and histograms and tested using a Shapiro–Wilk test to ensure a near-to-normal distribution. The relationship between heart rate and echocardiographic parameters was assessed using linear mixed models, accounting for repeated measurements within participants. Differences in participant characteristics were accommodated by incorporating participant id as a random effect. Increasing powers of heart rate were included as fixed effects as long as model fit increased significantly by the likelihood ratio test. *P*-values <0.005 were considered significant. Regression formulas and corresponding trend reductions with exact measurement values were derived from the outcome of these linear mixed models.

For continuous variables, mean (SD) or median (IQR) is presented as appropriate. Changes in echocardiographic measurements according to changes in heart rate are presented as mean (95% CI). For categorical variables, numbers (percentages) are presented. All statistical analyses were conducted using the computer software R, version 4.2.1, with packages stats and lme4, and Python, version 3.11, with packages pymer4 and pandas. The figures were produced using Python with packages matplotlib and seaborn.

### Ethics

The study was approved by the Regional Ethics Committee (project identification 7160), the Data Protection Officer at St. Olavs Hospital, and the Research Council at the Clinic of Cardiology, St. Olavs Hospital. Written consent was collected from each participant before inclusion.

## Results

### Study participants

Fifty participants were included, 52% females, age ranging from 27 to 88 years. Frequent comorbid conditions were hypertension, coronary artery disease, and paroxysmal atrial fibrillation (*Table 1*). Except for beta blockers (20%), none of the participants used anti-arrhythmic medications. Time since first pacemaker implantation ranged from 5 months to 16 years, with a mean of 6 (4) years.

**Table 1:** Clinical characteristics

Age, median (IQR) (years)	72 (19)
Female, *n* (%)	26 (52%)
Height, mean (SD) (cm)	172 (9)
BMI, mean (SD) (kg/m^2^)	28 (4)
*Cardiac diseases and risk factors*	
Paroxysmal atrial fibrillation, *n* (%)	10 (20%)
HFrEF (%)	1 (2%)
Hypertension, *n* (%)	19 (38%)
Coronary artery disease, *n* (%)	9 (18%)
History of myocardial infarction, *n* (%)	5 (10%)
Previous coronary intervention, *n* (%)	8 (16%)
Only PCI, *n* (%)	4 (8%)
Only CABG, *n* (%)	3 (6%)
Both PCI and CABG, *n* (%)	1 (2%)
Heart valve disease^a^, *n* (%)	2 (4%)
Diabetes mellitus, *n* (%)	5 (10%)
Reduced renal function, *n* (%)	0 (0%)
*Anti-arrhythmic drugs*	
Beta blocker, *n* (%)	10 (20%)
Other, *n* (%)	0 (0%)

Data are presented as median (IQR), mean (SD), or numbers (percentages). Total *n* = 50.

HFrEF, heart failure with reduced ejection fraction; PCI, percutaneous coronary intervention; CAGB, coronary artery bypass graft. Hypertension was defined as either noted in the hospital electronic patient journal, or use of anti-hypertensive medications. Heart valve disease was defined as moderate or severe stenosis or regurgitation in any valve, or prior valve replacement.

^a^(1) moderate aortic insufficiency and (2) mechanical mitral valve prosthesis.

At baseline echocardiographic acquisition, most participants were within normal ranges for LV volumes, systolic, diastolic, and RV function (*Table 2*). Only one participant had LVEF <40%, four had *E*/*e*′ > 14, and four had tricuspid annular longitudinal excursion <17 mm. Image quality was good or intermediate in most of the participants (*n* = 23 and *n* = 19, respectively). Eight participants had ≥2 LV segments with <50% visualization of the myocardial wall, indicating poor image quality. Still, all had at least one visible segment within each wall.

**Table 2:** Echocardiographic characteristics at baseline acquisition

GLS (%) by AFI	17.5 (3.3)
LVEF (%)	54 (7)
LVEDV (mL)	124 (34)
LVESV (mL)	58 (20)
MV *E*/*A* ratio	1.1 (0.9)
Mitral *E*/*e*′	9.1 (3.7)
TAPSE (cm)	2.2 (0.4), *n* = 49
TV *S*′ (cm/s)	10.9 (2.2), *n* = 47

Data are presented as mean (SD). Baseline parameters were calculated manually by an experienced cardiologist, in all patients unless otherwise stated. Total *n* = 50.

AFI, automated function imaging; MV, mitral valve; TAPSE, tricuspid annular longitudinal excursion; TV *S*′, tricuspid lateral annular systolic velocity.

### Heart rate range

At baseline, participants had either sinus rhythm (*n* = 39) or atrial paced rhythm (*n* = 11), with heart rates ranging from 50 to 87 beats/min. Mean heart rate at baseline was 64 (9) beats/min. Ten participants had the protocol terminated <120 beats/min, 10 participants terminated at 120 beats/min, 11 participants at 130 beats/min, whereas the upper target pacing rate of 140 beats/min was reached by 19 participants. The primary reason for terminating the pacing protocol was the development of Type 1 second-degree AV block (*n* = 28). In addition, two participants developed frequent ectopic beats, and one participant developed atrial fibrillation at a pacing rate of 100 beats/min, which was haemodynamically tolerable and self-terminating within minutes. Uncomfortable palpitations were very rare, even at the highest pacing rates.

Echocardiograms were obtained from nearly all participants in the range 80–100 beats/min, with 47 and 49 subjects at 80 and 100 beats/min, respectively. The complete protocol range of 50–140 beats/min was obtained in only two participants, mainly due to a limited number of participants at a baseline heart rate of below 50 beats/min. The heart rate range for analysis and figures was thus set to 60–140 beats/min, the whole range covered by eight participants.

### GLS at increasing heart rates

**Figure 1:**
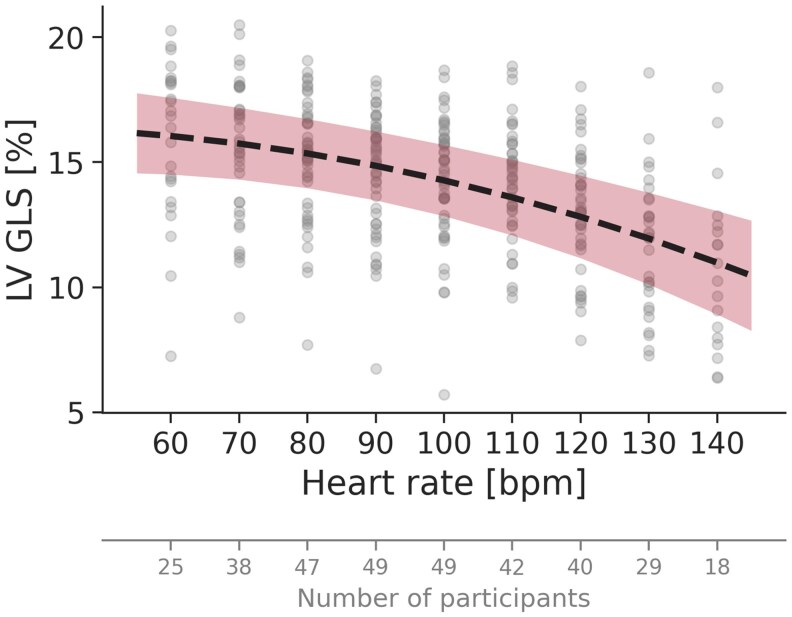
GLS with increasing heart rate. The dotted line represents the regression line from the GLS linear mixed model, and the coloured area indicates the corresponding 95% confidence interval.

With increasing heart rate, there was a reduction in GLS in almost all participants (94%). Estimated GLS decreased by 32% (95% CI: 19–44%), from 16.0% at 60 beats/min to 10.9% at 140 beats/min (*Figure 1*). The reduction followed a near-linear trend, but with a greater decrease in GLS for higher heart rate levels, consistent with a quadratic model (*P* < 0.001 compared to a linear model) (see Supplementary data online, *Table S1*). The strong relationship between changes in heart rate and GLS was supported by a highly statistically significant likelihood ratio test (*P* < 0.001).

### LVEF at increasing heart rates

**Figure 2:**
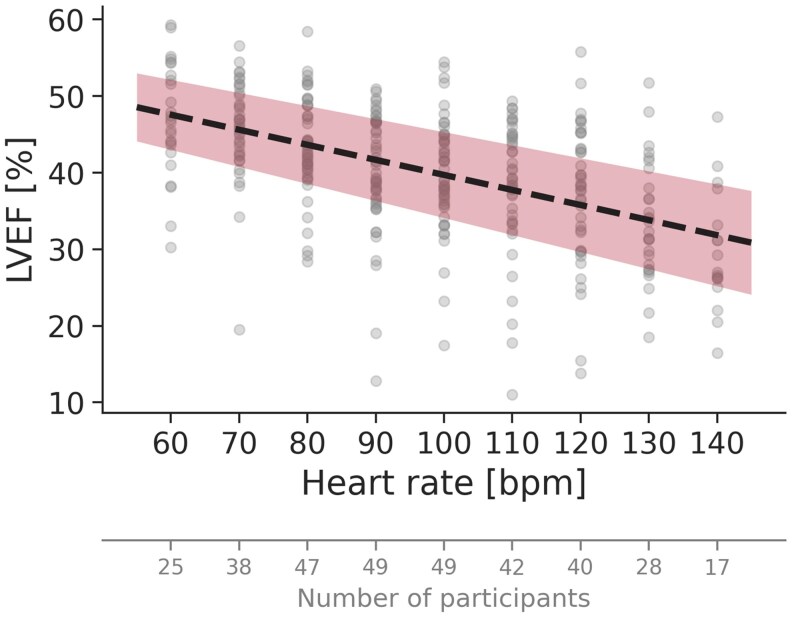
LVEF with increasing heart rate. The dotted line represents the regression line from the LVEF linear mixed model, and the coloured area indicates the corresponding 95% confidence interval.

With increasing heart rate, there was a reduction in LVEF in almost all participants (94%). Estimated LVEF decreased by 33% (95% CI: 19–47%), from 48% at 60 beats/min to 32% at 140 beats/min (*Figure 2*). The reduction followed a linear trend, with a mean decrease of 2.0% per 10 beats/min (see Supplementary data online, *Table S1*). The strong correlation between changes in heart rate and LVEF was supported by a highly statistically significant likelihood ratio test (*P* < 0.001).

### LV volumes and cardiac output at increasing heart rates

**Figure 3:**
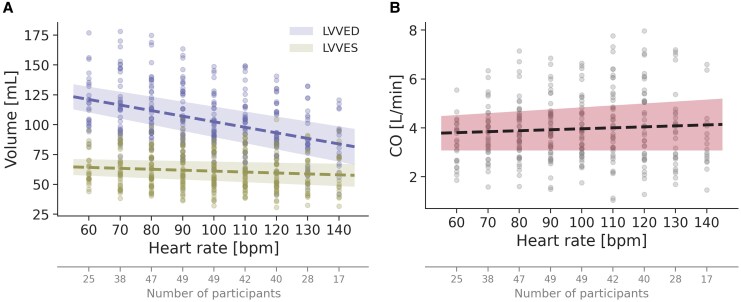
LVEDV and LVESV (*A*), and CO with increasing heart rate (*B*). The dotted lines represent the regression lines from the LVVED, LVVES, and CO linear mixed models, and the coloured areas indicate the corresponding 95% confidence intervals.

With increasing heart rate, there was a reduction in LVEDV and LVESV, although not proportional (*Figure 3A*). While estimated LVEDV decreased by 31% (95% CI: 19–43%) from 121 to 84 mL, estimated LVESV decreased by 10% (95% CI: −5% to 24%) from 64 to 58 mL, when increasing the heart rate from 60 to 140 beats/min. The reduction in LVEDV and LVESV both followed a linear trend, with a mean decrease of 4.7 and 0.8 mL, respectively, with every 10 beats/min (see Supplementary data online, *Table S1*). The strong correlation between LV volumes and changes in heart rate was supported by a highly statistically significant likelihood ratio test (both *P* < 0.001).

Corresponding to a more pronounced reduction in LVEDV compared to LVESV, CO remained relatively stable with increasing heart rate (*Figure 3B*). The minimal increase in CO followed a near-linear trend and is thus presented as a linear regression line (see Supplementary data online, *Table S1*). Still, the correlation between CO and changes in heart rate did not reach statistical significance, as indicated by the likelihood ratio test (*P* = 0.051).

### MAPSE at increasing heart rates

Estimated MAPSE decreased by 34% (95% CI: 23–45%), from 11.8 mm at 60 beats/min to 7.8 mm at 140 beats/min (see Supplementary data online, *Figure S1*). The reduction followed a linear trend, with a mean decrease of 0.5 mm per 10 beats/min (see Supplementary data online, *Table S1*). The strong correlation between changes in heart rate and MAPSE was supported by a highly statistically significant likelihood ratio test (*P* < 0.001).

### Feasibility and processing time

A total of 10 496 cardiac cycles were obtained. Of these, the DL method failed to analyse 335 cycles due to view misclassification, or insufficient segmentation or tracking quality, resulting in a total of 10 161 cycles for measurement calculations. Thus, feasibility for the automated measurements was 97%.

The processing time for automated measurements at baseline acquisitions was 1.3 (0.4) s per cardiac cycle, corresponding to an overall processing time of just 3.7 h for all analyses in the entire data set.

## Discussion

This study comprehensively examined the impact of increasing heart rate on key echocardiographic measurements of LV function and volume, utilizing a DL-based method for fully automated measurements to efficiently process the extensive dataset. We observed pronounced and clinically relevant decreases in all LV function and volume measures as heart rate increased. These findings underscore the necessity to incorporate heart rate into the clinical interpretation and reporting of echocardiographic measurements.

### Changes in echocardiographic measures induced by atrial pacing and increasing heart rate

This study demonstrates how stepwise increments in heart rate led to a highly significant reduction in GLS, LVEF, and LV volumes, with either near-linear or linear relationships. MAPSE followed a similar trend. A near-to-normal activation of the left ventricle was achieved by selecting a study population with isolated sinus node dysfunction, preserved AV-conduction, and permanently implanted programmable pacemakers, enabling controllable heart rate alterations using atrial pacing mode only. Unlike pharmacologically or exercise-induced heart rate alterations, involving multiple adaptive mechanisms—such as increased preload from augmented venous return (via skeletal muscle and respiratory pumps, and sympathetic venoconstriction), enhanced contractility through sympathetic stimulation, and reduced afterload due to systemic vasodilation^15^—atrial pacing isolates the effect of heart rate by minimizing neurohumoral influences on the cardiovascular system. Although this approach does not fully replicate the physiological response to increased metabolic demand, it offers a controlled setting to assess the direct impact of heart rate on echocardiographic measurements of cardiac function. Such distinction may be particularly valuable when evaluating cardiac performance in patients with tachyarrhythmias occurring independently of physical stress.

Prior studies utilizing atrial pacing, predominantly conducted in the 1970s and 1980s, suggested similar changes as in the present study, demonstrating reductions in LVEDV, LVESV, and LVEF with increasing HR.^2–4^ However, their clinical interpretation was restricted by small sample sizes, few observations, limited heart rate ranges, or patient populations with severe heart disease. Since then, newer echocardiographic techniques have emerged, including GLS, which offers valuable diagnostic and prognostic value and has demonstrated greater reliability than traditional measures such as LVEF.^16,17^ A more recent study investigated the relationship between HR and GLS, but their cohort primarily consisted of patients with cardiac disease, with 84% diagnosed with chronic heart failure, limiting the generalizability of the findings.^18^ In contrast to earlier studies, the present study employed state-of-the-art DL-based measurements for accurate and reproducible measurements across multiple heart cycles and pacing levels. We included a heterogeneous cohort representative of patients in clinical practice, adding new insights into HR-dependent changes in modern echocardiographic parameters.

While our study demonstrated a highly significant decrease in echocardiographic measures of LV function and volumes with increasing heart rate, a previous study by Fredholm *et al.* found that heart rate alterations alone had no effect on echocardiographic LV strain when maintaining preload.^19^ In contrast, increased preload alone led to a 20% reduction in LV strain. While Fredholm *et al.* controlled preload by volume substitution throughout atrial pacing, enabling separate assessment of heart rate and preload effects, we altered heart rate without any volume compensation. Thus, as increase in heart rate reduces filling times, heart rate and preload were not independent factors in our study, and we could not distinguish changes in strain from changes in preload. However, the findings by Fredholm *et al.* suggest that the observed changes in our study primarily reflect changes in preload.

Even though atrial pacing reduces neurohumoral influences on the cardiovascular system, heart rate remains a principal determinant for LV diastolic filling time. Diastolic filling time and preload are consequently reduced with increasing heart rate. Given these changes, one could argue that heart rate will influence echocardiographic measurements in clinical practice, particularly diastolic parameters. In contrast, systolic ejection time is less influenced by increasing heart rate.^20^ This physiological phenomenon is reflected in the presented discrepancy between changes in LVEDV and LVESV, reductions of 31% and 10%, respectively, and is consistent with previous reports.^2,4^ Still, the present findings indicate an even more pronounced discrepancy, showing a decrease of only 0.8 mL in LVESV, compared to 2.0 and 1.3 mL per 10 beats/min in prior studies.^2,4^ This discrepancy resulted in a relatively stable CO throughout all heart rates in our study, which is physiologically sound as there was no increased systemic metabolic demand.

### Automating analyses of large data

This study demonstrated the application of DL to automate echocardiographic measurements across a large dataset. Instead of relying on manual or semi-automatic measurements, DL was utilized to increase time-efficiency, eliminate observer-related variability, and reduce random and systematic noise by averaging measurements over multiple cardiac cycles. This approach enabled a more comprehensive exploration of echocardiographic measurements across a wide range of heart rates than previously feasible, with a total processing time of <4 h. In comparison, an estimated time consumption of ∼5.5 min per cardiac cycle using conventional manual measurements would result in a total analysis time of ∼930 h just for LVEF and LV volumes.^9^ GLS calculations, not included in this time estimate, would easily double this analysis time estimate to approximate 1 year of dedicated manual measurements.

### Clinical implications

The findings of this study highlight the profound impact of heart rate on key echocardiographic measures of LV function and volumes, emphasizing the necessity of accounting for heart rate in clinical echocardiography. These findings should be taken into consideration by both acknowledging a likely decrease in LV function measures and volumes with higher heart rates, and by actively assessing heart rates when comparing echocardiographic data across time points within the same patient. Furthermore, our regression formulas (see Supplementary data online, *Table S1*) and figures (*Figures 1–3*) indicate the range of values that can be expected for GLS, LVEF, and LV volumes at different heart rates using the DL-based method for similar patient cohorts, as well as the expected change in these parameters with changes in heart rate alone. This approach could be valuable for patient follow-up, helping to discern whether changes in LV measurements indicate genuine alterations in cardiac function or merely could be a consequence of heart rate variations. This distinction is crucial for patients whose echocardiographic changes may influence treatment decisions, such as in cardio-oncology. In our population, the criteria for possible cardiotoxicity (more than 10% change in absolute LVEF or more than 15% change in relative GLS) will be met by a heart rate change from 60 to 110 beats/min.^21^ These findings underscore the importance of considering heart rate as a contributing factor when interpreting changes in LV function.

### Strengths and limitations

A strength of this study is the application of controlled heart rate variation by atrial pacing, preventing changes in LV contraction patterns and thus minimizing external variables. Furthermore, averaging multiple cardiac cycles at each heart rate level minimizes the noise introduced by random and systematic beat-to-beat measurement variability, revealing actual changes between heart rate levels even at an individual level. To further reduce operator-related variability, all acquisitions were performed by only two expert clinicians. Further, the diversity in patient categories enhances the applicability of the findings to real-world scenarios, providing valuable insight into echocardiographic evaluations within a broader and more realistic clinical context than in previous research.

The study still has potential limitations. Even though atrial pacing enables a near-to-normal activation of the left ventricle, heart rate changes introduced in this study do not accurately replicate the physiological fluctuations seen in clinical practice, triggered by factors such as physical activity, adrenergic stress, sepsis, or pharmacological influence. Additionally, the echocardiographic acquisitions at each pacing level were initiated immediately after stabilization of the target heart rate, possibly influencing the measurements. One potential confounding factor could be due to the Bowditch effect, which describes how an increase in heart rate enhances myocardial inotropy due to an increase in intracellular calcium levels at higher stimulation frequencies.^22^ If calcium homeostasis had not fully stabilized during acquisitions, the resulting inotropic response may have been somewhat lower than at a sustained, steady-state heart rate.

Moreover, the study population comprised 50 participants, with only two individuals exhibiting the full range of heart rates from 50 to 140 beats/min. Most of the participants were elderly, and a substantial proportion had concomitant cardiac diseases and/or risk factors. The estimated means for LV parameters may consequently differ from those obtained in a younger population without comorbidities. Still, it is reason to believe that this study population is representative of patients undergoing echocardiography in routine clinical practice. Furthermore, frame rate was kept constant throughout the echocardiographic examination for each patient, resulting in a lower relative sampling of measurements at higher heart rates. However, a synthetic down-sampling at baseline resulted in no significant change in measurement outcomes, suggesting that the undersampling was less impactful than the effect of physiological changes with increasing heart rate. A slight discrepancy was observed between GLS values from the DL method (16.0% at 60 beats/min) and manual measurements (17.5% at 60 beats/min). This likely reflects a minor bias between measurement methods, as noted in prior studies,^8,16^ emphasizing the importance of focusing on the measurement trends across heart rates rather than exact values.

### Future research

This study should inspire further research into how heart rate influences both systolic and diastolic function as assessed by echocardiographic measurements. Such research should explore different pacing strategies, as well as other means of inducing heart rate alterations, in order to validate and extend the present findings. Moreover, by demonstrating the capacity of fully automated DL-based measurements to process large datasets with exceptional speed, this study highlights the broader potential for artificial intelligence to support research tasks previously considered unattainable. Continued development and validation of automated methods will be essential to achieve a more comprehensive assessment of cardiac function and to delineate the relative contributions of systolic and diastolic changes.

## Conclusion

Common echocardiographic measures of LV function and volume, including GLS, LVEF, LVEDV, and LVESV, are significantly influenced by heart rate. These findings highlight the need to incorporate heart rate in the reporting and clinical interpretation of echocardiographic measurements. This study further demonstrates the potential of DL to advance cardiovascular research by enabling rapid and reproducible analyses in large datasets, previously unachievable due to the inherent constraints of manual measurements.

## Supplementary Material

qyaf163_Supplementary_Data

## Data Availability

The data collected during this study will be made available upon reasonable request to the corresponding author.

## Lead author biography

Ada Woelfert is a fifth-year medical student and a participant in the medical student research programme. Her research focuses on echocardiography and the application of artificial intelligence for automated cardiac assessment. After graduating, she plans to finalize her PhD before beginning clinical training.
