# Supplementary material for: The impact of heart rate on echocardiographic measures of left ventricular function: novel insights facilitated by deep learning
Source: Eur Heart J Imaging Methods Pract. 2025 Dec 24;4(1):qyaf163. doi: 10.1093/ehjimp/qyaf163 (PMC12798807; doi:10.1093/ehjimp/qyaf163)
Supplement: qyaf163_Supplementary_Data [file qyaf163_supplementary_data.pdf]

Supplemental Material

Figure S1: MAPSE with increasing heart rate. The dotted line represents the regression line from the MAPSE linear mixed model, and the coloured area indicates the corresponding 95% confidence interval.

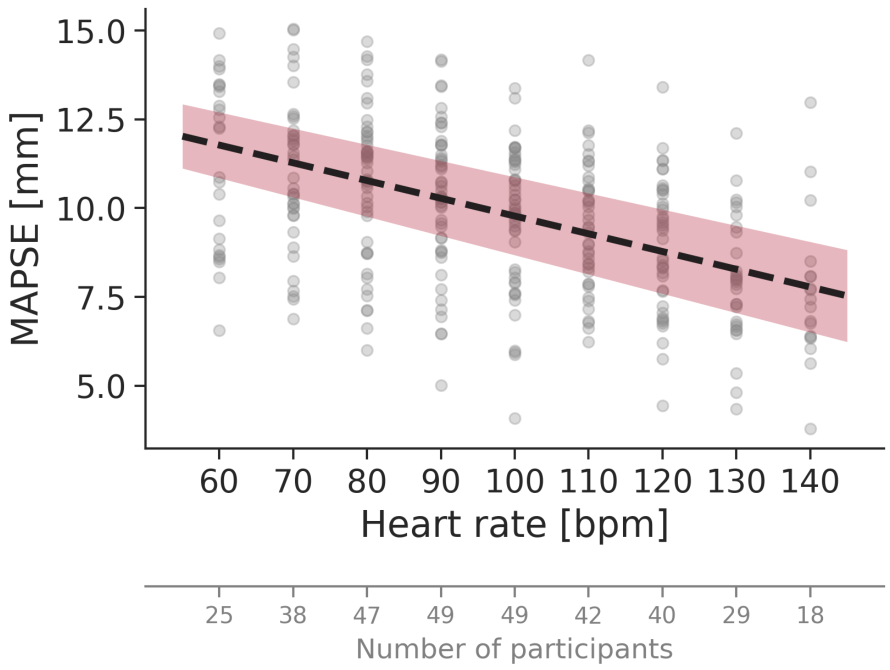

Table S1. Regression Formulas from Linear Mixed Models

GLS (%)	20.3913 – 0.0611 × HR – 0.0005 × (HR – 97.7448)^2
LVEF (%)	59.3695 – 0.1966 × HR
LVEDV (mL)	148.9649 – 0.4652 × HR
LVESV (mL)	68.8559 – 0.0779 × HR
CO (L)	3.5681 + 0.0040 × HR
MAPSE (mm)	14.7700 – 0.0500 × HR

GLS: global longitudinal strain; LVEF: left ventricular ejection fraction; LVEDV: left ventricular end-diastolic volume; LVESV: left ventricular end-systolic volume; CO: cardiac output; MAPSE: mitral annular plane systolic excursion; HR: heart rate.
